# Efficiency of Teeth Bleaching after Regenerative Endodontic Treatment: A Systematic Review

**DOI:** 10.3390/jcm10020316

**Published:** 2021-01-16

**Authors:** Irini Fagogeni, Tomasz Falgowski, Joanna Metlerska, Mariusz Lipski, Maciej Górski, Alicja Nowicka

**Affiliations:** 1 Doctoral Studies of the Faculty of Dentistry, Pomeranian Medical University in Szczecin, 70-111 Szczecin, Poland; irini.fagogeni@gmail.com (I.F.); asimoza@wp.pl (J.M.); 2 General, Minimally Invasive and Gastrointestinal Surgery Department, Pomeranian Medical University in Szczecin, 70-111 Szczecin, Poland; falgowski.tomasz@gmail.com; 3 Department of Preclinical Conservative Dentistry and Preclinical Endodontics, Pomeranian Medical University in Szczecin, 70-111 Szczecin, Poland; lipam@pum.edu.pl; 4 Department of Conservative Dentistry and Endodontics, Pomeranian Medical University in Szczecin, 70-111 Szczecin, Poland; maciek@dentist.szczecin.pl

**Keywords:** bleaching, carbamide peroxide, hydrogen peroxide, regenerative endodontic treatment, revascularization, sodium perborate

## Abstract

The aim of this review is to evaluate of effectiveness of bleaching procedures used to treat discolored teeth subsequent to regenerative endodontic procedures (REPs) based on the review of in vitro and in vivo studies. This literature review was carried out according to the PRISMA guidelines. Four databases (PubMed, Scopus, the Cochrane Library, and Web of Science databases) were searched electronically, until 30 January 2020 without a year limit. The quality of studies was assessed using a modified methodological index for non-randomized studies. After analyzing 1405 studies, 6 in vitro and 9 in vivo studies were eligible for this review. In in vitro studies, effectiveness of bleaching was assessed in teeth discolored by antibiotic pastes, blood, and barrier materials in various combinations. In all analyzed studies, bleaching was effective in teeth discolored by antibiotic pastes as well as by blood and barrier materials. Of 26 treated teeth in the in vivo studies, 17 teeth were bleached successfully. In six cases, there was improvement of the shade. In three cases, bleaching was not sufficient. Bleaching material, techniques, and times differed between studies. Whitening of discolored teeth after REPs is achievable. However, to establish precise guidelines, further long-term clinical studies should be performed.

## 1. Introduction

Regenerative endodontic procedures (REPs) are a recently expanding field in endodontics. They are “biologically based procedures designed to physiologically replace damaged tooth structures” [1]. Regeneration of damaged dentin and root structures, as well as the pulp-dentin complex, are fundamental goals of these procedures [1]. REPs are increasingly applied in immature permanent teeth with pulpal necrosis (with or without apical periodontitis) as an alternative treatment option to apexification. Unlike traditional apexification, physiological root development and maturation represent the greatest advantages of this method [2]. However, some studies report the occurrence of tooth discoloration subsequent to REPs [3,4], which is an unfavorable outcome of these procedures [5,6]. The potential causes of observed discoloration are respectively: intracanal medicaments, distribution of blood products, and compositions of barrier materials used in REPs.

Teeth discoloration may negatively impact the quality of life in young patients and their families [7], especially if the problem concerns anterior teeth. To minimize the risk of discoloration, placing of triple antibiotic paste (TAP) containing minocycline (TAPM) below the cemento-enamel junction and sealing the pulp chamber with a dentin bonding agent should be considered [8]. However, authors have stated that this recent procedure could not completely eliminate the problem [9,10].

Masking the discoloration with composite resin veneer or internal bleaching are the treatment options used to reduce or eliminate tooth discoloration after REPs [11]. Simple, viable, and minimally invasive procedures should be considered as a treatment of choice. The ideal option would be dental bleaching. However, the 2012 Cosmetic Products Safety Amendment Regulations allowed the use of <0.1% hydrogen peroxide and other compounds or mixtures that release hydrogen peroxide on a group of patients under 18 years old [12,13]. This is problematic situation faced by dentists, when there are indications for performing bleaching, but the procedure is illicit [13]. Fortunately, a new regulation in the General Dental Council’s Position Statement on Tooth Whitening currently available on their website claims that products containing or releasing between 0.1% and 6% hydrogen peroxide cannot be used in young patients under 18 years old, but the exception is when the whitening is aimed at treating or preventing disease [14].

Hydrogen peroxide is an active ingredient in bleaching materials [15]. It can penetrate the dentin and releases oxygen, which breaks double bonds of the organic and inorganic compounds inside dental tubules [15,16]. Hydrogen peroxide is applied directly or might be formed as a result of a chemical reaction from sodium perborate or carbamide peroxide [15].

Whitening improves the color of the discolored tooth. However, not always to a sufficient degree [17]. There are currently no precise guidelines for dental bleaching of discolored teeth subsequent to REPs. The bleaching procedure differs across the currently available studies. The aim of this review was to identify the effectiveness of teeth whitening after REPs.

## 2. Materials and Methods

### 2.1. Review Questions

**Figure 1:**
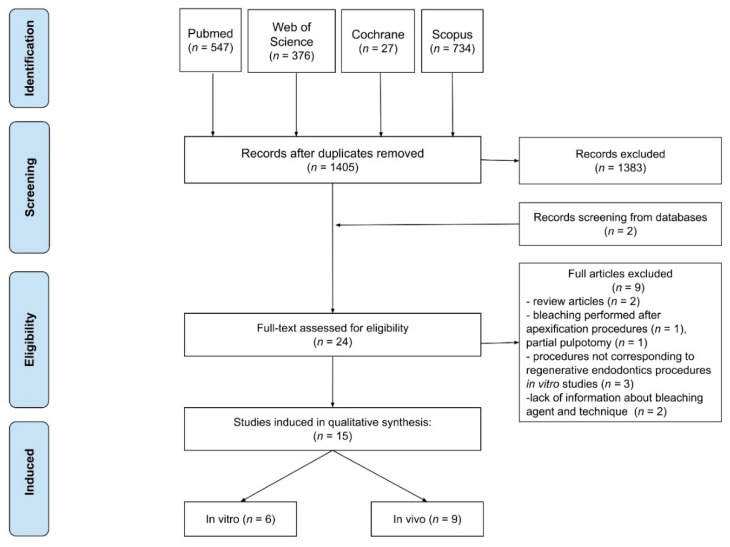
Preferred Reporting Items for Systematic Reviews and Meta-analyses (PRISMA) flow diagram of the search strategy.

The literature review was performed in accordance with the Preferred Reporting Items for Systematic Reviews and Meta-analyses (PRISMA) standards (Figure 1) [18]. This review aims to find an answer for the questions listed below:

1. Is whitening of discolored teeth after REPs effective?
2. What kind of bleaching techniques should be used after REPs?
3. What kind of bleaching agents should be applied in bleaching techniques after REPs?
4. How long should bleaching last?

### 2.2. Search Strategy and Study Selection

Four databases (PubMed, Scopus, the Cochrane Library, and Web of Science databases) were searched electronically by two independent reviewers (I.F and T.F) for publications involving bleaching of teeth discolored after REPs. Publications were searched without a year limit. The last search was conducted on 30 January 2020. The search phrases are presented in Table 1. After removing duplicates all titles and abstracts were examined.

Publications were extracted based on the inclusion criteria listed below:

1. In vitro and in vivo studies that evaluated bleaching of tooth discoloration after REPs.
2. Publications in which bleaching material was placed in the tooth tissues.

### 2.3. Quality Assessment

The quality of in vitro and in vivo studies was assessed using a modified methodological index for non-randomized studies (MINORS) [19]. This index includes twelve items: the first eight items pertain to non-comparative studies and an additional four concern comparative studies. Search results of in vivo studies including case series and case reports. The risk of bias assessment tool to evaluate the methodological quality of case reports was not developed [20]. To show an overview and standardize different types of studies including case reports and case series a modified MINORS scale was used analogous to that of Benetti et al. [21]. The modified MINORS scale items were as follows: clear aim, clear REPs protocol, clear bleaching protocol, prospective collection of data, justification of sample size, follow-up period appropriate to the aim of the study, endpoints appropriate to the aim of the study, blinded analysis, an adequate control group, contemporary groups, baseline equivalence of groups, and adequate statistical analyses. The items were scored: 0, not reported; 1, reported but inadequate; and 2, reported and adequate. All twelve items were used to assess in vitro studies, of which the first eight items were used to evaluate the in vivo studies. The ideal score for comparative studies is 24 and for non-comparative studies 16 [19]. The classification of quality of the in vivo studies was made on according to that of Elkhadem et al. [20] into poor (0–5), fair (6–10), and good (11–16) and analogously a similar classification was used for in vitro studies: poor (0–8), fair (9–16), and good (17–24). The results of each item, total score, and study quality are presented in Table 2 and Table 3 for the in vitro and in vivo studies, respectively.

## 3. Results

After analyzing 1405 studies retrieved from the query of all databases, six in vitro and nine in vivo studies were qualified on using the PRISMA criteria (Figure 1). Two of the included studies were searched from additional sources [11,30]. Articles excluded were studies in which the procedure did not correspond to REPs in in vitro studies [33,34,35]; studies where bleaching was performed after apexification [36] or partial pulpotomy [37] in in vivo studies; studies lacking information about bleaching agent and technique [38,39]; and review articles [4,40].

### 3.1. Results of In Vitro Studies

**Figure 2:**
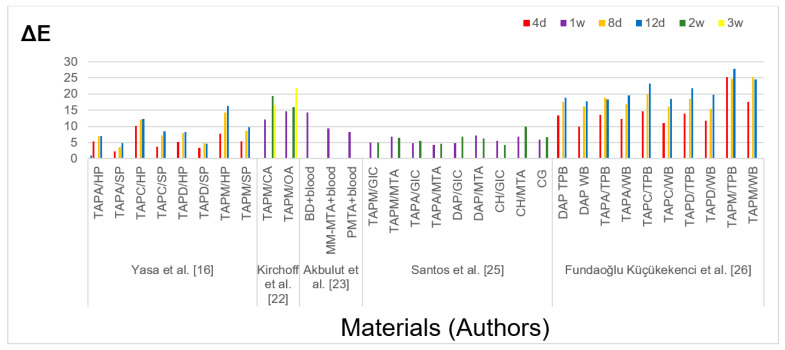
Delta E values defining the color changes of the tooth induced by bleaching materials reported in in vitro studies. Abbreviations: BD, Biodentine; CA, closed apices; CG, control group; d, day; DAP, double antibiotic paste; GIC, glass ionomer cement; HP, hydrogen peroxide; MTA, mineral trioxide aggregate; OA, open apices; PMTA, Pro Root MTA; SP, sodium perborate; TAPA, triple antibiotic paste with amoxicillin; TAPC, triple antibiotic paste with cefaclor; TAPD, triple antibiotic paste with doxycycline; TAPM, triple antibiotic paste with minocycline; TPB, thermo/photo bleaching; w, week; WB, walking bleaching; WMTA, white MTA.

In six in vitro studies, both bovine teeth [16,25] and human teeth [22,23,24,26] were bleached. Bleaching was carried out in discolored teeth after the application of antibiotic pastes, blood, and barrier materials in various combinations. Sodium perborate [16,22,24], hydrogen peroxide [16,23,26], and carbamide peroxide [25] were used as bleaching agents. Bleaching material was placed inside [16,22,23,24,25,26] or inside and outside [25] dental crowns. The authors evaluated color changes using spectrophotometric measurements [16,22,23,24,25,26] which allows to obtain the CIELAB color coordinates for a quantitative evaluation of color changes and based on the VITA Classical A1–D4^®^ Shade Guide, a standard and reference system used worldwide in tooth shade determination [25]. In studies in which antibiotic pastes were used, a tooth stained with TAPM bleached more than a tooth stained with TAP with doxycycline (TAPD) and amoxicillin (TAPA) using both 35% hydrogen peroxide and sodium perborate [16,26]. In studies in which TAP with cefaclor (TAPC), blood and barrier material such as Biodentine (Septodont, Lancasted, PA, USA), MM-MTA, (Micro Mega, Besancon Cedex, France), and ProRoot MTA (Dentsply, Tulsa, OK, USA) were used and bleaching was performed with 35% hydrogen peroxide, the group treated with Biodentine bleached significantly more than in other groups [23]. Information extracted from qualified in vitro studies including number and teeth type, intracanal medicament or/and coronal barrier, discoloration, bleaching material and technique, the measurement method, and time as well as bleaching effect are listed in Table 4. Figure 2 presents delta E values from studies, which included its value among the results. 

### 3.2. Results of In Vivo Studies

Nine in vivo studies related to bleaching after REPs were identified in this review, seven of which were case reports [9,11,27,28,29,30,31] and two case series [17,32]. Of the 26 treated teeth, 23 were anterior teeth [9,11,17,27,28,30,32] and three involved premolar teeth [29,31]. Bleaching was performed in teeth in which discoloration was caused by intracanal pastes (e.g., TAP with clindamycin [TAPK], TAPM, TAPA, Odontopaste, double antibiotic paste [DAP], calcium hydroxide [CH] and chlorhexidine gel) and barrier materials (e.g., mineral trioxide aggregate [MTA], white MTA [WMTA], grey MTA [GMTA], and Portland Cement [PC]). Internal, external and both of techniques were carried out to bleach discolored teeth. In the studies discolored teeth were bleached with different bleaching agents: sodium perborate [9,11,27,28,31], hydrogen peroxide [29], and carbamide peroxide [32]. Two authors used the combination of sodium perborate and hydrogen peroxide [17,30]. Sodium perborate was the most frequent bleaching agent used in the in vivo studies. Whitening improved the color of the discolored teeth [11,28,29,30] both without [32] and with patient satisfaction [27,31]. However, teeth did not always return to their original shade [9]. Of all the analyzed studies, three cases did not bleach sufficiently [17]. Table 5 contains details from in vivo studies including patient ages, tooth type, injury, preoperative diagnosis, medicaments applied in REPs, discoloration, bleaching material and methods, as well as bleaching outcome.

A statistical analysis could not be performed because of different parameters used by reviewed studies.

## 4. Discussion

This review investigated the efficiency of bleaching tooth discoloration after REPs. All qualified studies were divided into two groups, in vitro and in vivo studies, and were discussed separately.

### 4.1. Analysis of In Vitro Studies

Most of the analyzed studies evaluated the whitening effect of tooth discoloration caused by disinfectant pastes [16,22,24,26] because root canal disinfection is the most important and initial stage in the revascularization procedure in which discoloration could be observed. In the in vitro studies included in this analysis where TAPM was used, coronal discoloration was observed more often than when other disinfectant pastes were applied [16,25,26]. A tooth stained by TAPM whitened more compared to teeth discolored by TAPD, TAPA, TAPC, or DAP [26]. Yasa et al. [16] found that the whitening effect was greater when teeth were discolored by TAPM and TAPC compared to groups with TAPD and TAPA. However, the number of teeth in the groups was smaller (*n* = 5) than in the study by Fundaoğlu Küçükekenci et al. [26] and there was no information on the concentration of TAPC used [16].

Effective bleaching was achieved using 35% hydrogen peroxide [16,26], sodium perborate [16,22,24], and 37% carbamide peroxide [25]. The whitening effect of 35% hydrogen peroxide was greater than that of sodium perborate [16]. The bleaching effect of 35% hydrogen peroxide exceeded the perceptibility threshold from the 4th day of the evaluation and increased over time [26]. The highest bleaching effect was noticed on the 12th day [26], but another study showed no statistically significant difference (P = 0.175) between the 8^th^ and 12th day of measurements [16]. The number of teeth in the test groups differed between studies [16,26]. Nd-YAG laser irradiation on a 35% hydrogen peroxide increased the efficacy of internal bleaching, but there was no significant difference between the walking bleach technique and the thermo/photo bleaching technique (P = 0.19) [26]. Akbulut et al. [23] assessed the effectiveness of whitening teeth in which discoloration was induced respectively by TAPC, blood, and barrier materials, such as Biodentine, MM-MTA, or ProRoot MTA. In this study, 35% hydrogen peroxide was applied over the coronal barrier. The group with teeth discolored by Biodentine was bleached significantly more than with MM-MTA and ProRoot MTA, while no statistically significant difference was observed between specimens in groups treated with MM-MTA and ProRoot MTA [23]. The differences could be related to the composition of barrier materials such as calcium silicate cements. Biodentine contains zirconium oxide as a radiopacifying component, while ProRoot MTA and MM-MTA contained bismuth oxide, which is associated with tooth discoloration. Calcium silicate cements with zirconium oxide exhibited less discoloration [41]. This may be attributed to it being a finer sized particle [42] and, therefore, its effect on calcium silicate material diffusion into dentin tubules [43]. Moreover, Biodentine has also a highly specific surface area [42], which possibly increases the effectiveness of the bleaching agent [23]. However, overoxidation of bismuth oxide contained in ProRoot MTA and MM-MTA could result in discoloration [41] and thus, may lead to a reduction in whitening efficiency.

Sodium perborate is effective as a whitening agent and improves discoloration caused by TAPM [22,24]. Kirchhoff et al. [22] studied the ability of sodium perborate to bleach stained teeth with open and closed apices. The results indicated that the group with open apices bleach was similar to groups with closed apices, which is an interesting result, especially since younger teeth have potentially wider dentinal tubules [44]. Iriboz et al. [24] observed that there were no significant differences in bleaching on stained teeth with TAPM and minocycline paste when sodium perborate was used without or with activation by heat from a hand instrument, using an ultrasonic instrument for 30 s at a frequency at 29 kHz or 60 s at a frequency at 28 kHz. Increasing the temperature of bleaching agent with a heated hand instrument has been historically described [45], although excessive heating may damage the dental pulp in vital tooth bleaching [46] and increases the risk of external cervical resorption in non-vital tooth bleaching [47]. Currently in clinical procedures bleaching agents’ activation by heat from a hand instrument is not performed. Santos et al. [25] bleached specimens using 37% carbamide peroxide gel. Bleaching agent was placed two times with 1-week interval inside and outside the dental crown for 45 min. In the same group no difference was found between first and second bleaching. It may suggest that only one session of bleaching is effective to improve color shade.

### 4.2. Analysis of In Vivo Studies

Regenerative endodontic procedures involved all treated teeth, of which only one was completed in a single visit [31]. In analyzed studies, a change in color was noticed some time after the disinfectant paste was placed or post-treatment. In two of the included studies, grey and blue-greyish discoloration was observed six weeks after placing TAPM [9,27]. TAPM was most frequently used as an intracanal disinfectant [9,17,27,28,30]. However, other materials were also used (e.g., TAPK, Odontopaste, TAPA, DAP, CH, and 2% chlorhexidine gel). Parthiban et al. [30] noticed mild discoloration 28 days after TAPM placement and blue-greyish discoloration three months post-treatment. In other cases, post-treatment discoloration time occurrence varied between studies. It is worth mentioning that not all studies contained information about the color of the discoloration.

The research methodology differed between studies. Bleaching was performed after one week [9], 39 weeks [27], 3 months [30], 39 months [11], and 58 months [31]. Some authors [17,28,29] did not include information about the post-treatment time before starting whitening in their studies. Bleaching was carried out using different techniques: internal, external, or both of these techniques.

Internal bleaching in which the bleaching material was placed into the pulp chamber was similar to the walking bleach technique carried out for non-vital teeth. Although vital pulp-like tissue is formed in root canal following REPs, the coronal pulp space is empty. This allows using the non-vital teeth bleaching technique to treat discolored teeth after REPs because cervical sealing material protects the new vital pulp-like tissue from the bleaching agent [25].

Bleaching techniques were carried out in asymptomatic patients immediately after the regeneration procedure had been performed in the teeth in which the hard tissue bridge did not manage to form [9] or after some time from bleaching when continued lateral wall thickening [11], periapical healing and maturation of the root apex [31] was observed. Kim et al. [9] performed bleaching one week post-treatment. The barrier material (MTA) was left in canal and the bleaching agent was placed over the cervical sealant with glass ionomer cement (GI). Eight months after bleaching the radiograph showed evidence of continuation of apical closure. Despite the only barriers separating the newly formed pulp-like tissue were MTA and GI, respectively, revascularization procedures were successful, which was a very meaningful result, especially since bleaching materials may damage the pulp tissue and cause pulp inflammation [21]. However, it is worth noticing that Tsujimoto et al. [48] using a scanning electron microscope observed changes in surface structure of MTA after application of hydrogen peroxide as a bleaching agent. Discovered structural alterations described as e.g., globular structures, woodpecker holes and creases were dependent on hydrogen peroxide concentration and may predispose to microleakage occurrence. Although the findings suggest that MTA is an insufficient barrier against tooth bleaching, an in vitro study of 2-mm intra-orifice barriers of GMTA, WMTA and GI showed similar coronal leakage in all tested materials [49]. The studies with a fluid transport model [49] and protein leakage test [50] showed that cervical sealing material significantly reduces leakage even when bleaching agent is in use. Therefore, seeing the fact that sealing materials are highly required as they reduce the possibility of resorption further investigation is essential. The formation of the dentin bridge underneath the barrier material could act as an additional biological seal. The formation of the dentin bridge underneath the barrier material could act as an additional biological seal. Additionally, studies that analyzed the thickness of the dentin bridge formation after direct pulp capping showed that the mean thicknesses of the hard-tissue dentin bridge depended on the material used [51].

Unfortunately, there are risks associated with internal bleaching of non-vital teeth, such as weakening of the physical properties of dental hard tissues, penetration of the bleaching agent in the dentinal tubules, dental fracture during treatment, and the most serious, external cervical root resorption [52]. No information on the above-mentioned complications was included in the in vivo studies analyzed. To avoid cervical root resorption in the treatment of non-vital tooth discoloration, a cervical barrier should be placed to prevent diffusion of the bleaching agent throughout the dentinal tubules [53]. Similar to this technique, the authors in the analyzed studies also applied cervical sealing materials such as GI [9,11,30,32], Cawit W (3M, St Paul, MN, USA) [31], or Coltosol (Coltene Whaledent, Mahwah, NJ, USA) [28] which was placed in cases when barrier material (e.g., MTA, WMTA, PC) was removed [31] or left [9,11,28,32] in the canal. Some authors did not report on the cervical sealing material [17,27,29].

Among the widely used bleaching materials such as carbamide peroxide, hydrogen peroxide, and sodium perborate, the last one was mainly used. Sodium perborate has been classified as carcinogenic, mutagenic, or toxic to reproduction (CMR substances) and its use is prohibited in Europe (Cosmetics Regulation 1223/2009) [54]. In analyzed studies, sodium perborate was mixed with hydrogen peroxide [17,30], distilled water [9,28,31], sterile water [11], or saline [27] and was placed in the pulp chamber. McTigue et al. [17] also applied a cotton pellet saturated with Superoxol (Sultan Healthcare, Hackensack, NJ, USA) for 3 min. The duration of whitening time varied from one to three weeks. The bleaching agent was placed in the pulp chamber once [11,17,27,31], twice [28], and three times [9] depending on the study. Only in one study was calcium hydroxide placed in the access cavity after bleaching for one week [30] and in one study was the evaluation of color changes made using a digital spectrophotometer [28].

Antov et al. [32] showed three cases in which 10% carbamide peroxide was used in the bleaching procedure. In two cases, two weeks of internal/external bleaching was performed using vacuum formed bleaching trays with a reservoir over the labial surface of the bleached teeth. Before bleaching reduction of the barrier material and cervical sealing with GI were performed. Bleaching showed a satisfactory final result in the first case and minimal improvement of shade in the second case. In the third case, because of the lack of radiographic evidence of Portland cement and calcific barriers, and therefore, the risk of damage to revitalized tissue upon reduction of cement, two weeks of external bleaching was performed. Shade was improved but the patient was not fully satisfied with the result, which was why a direct composite veneer was provided.

From 26 treated teeth 17 teeth were bleached successfully [11,17,28,29,32]. In six cases, there was an improvement of the shade [9,27,30,31,32] of which, in one case presented by Antov et al. [32], the effect was minimal and, in a case presented by Kim et al. [9], the tooth did not return to its original shade. In three cases bleaching was not sufficient [17].

The clinicians who perform REPs should be aware of the high-risk of post-treatment discoloration [32] and all procedures ought to be performed with minimal risk of potential discoloration. If the discoloration appears, bleaching should be considered ultimately to improve shade or in the transitional period before more invasive procedures were planned such as porcelain veneers or crowns.

There are no exact guidelines for dental bleaching of discolored teeth after REPs. There are no randomized trials on this topic. However, based on analyzed studies a summary of the current bleaching procedure was presented below.

Three different bleaching techniques were used in the analyzed studies: internal, internal-external, and external bleaching technique, of which internal bleaching was the most frequently used method in an asymptomatic patient [9,11,27,30,31]. It is worth mentioning that internal bleaching technique is commonly used in non-vital teeth and analyzed studies provide insufficient information about follow-up and long-term observations after this method was applied in terms of its effect on the outcome of teeth regeneration procedure. The barrier material was usually left in the canal [9,11,28,32], which may affect the additional seal and minimize the risk of damage to dentin bridge or revitalized pulp. Cervical sealing with GI cement was frequently applied to the barrier material [9,11,32]. The bleaching agent was placed inside the dental crown analogously to a non-vital tooth bleaching technique [9,11,17,27,28,29,30,31]. As a bleaching agent, three different substances were used: hydrogen peroxide, sodium perborate, and carbamide peroxide. It is impossible to conclude which bleaching agent should be recommended owing to high variability in the investigated studies—different bleaching agents, its concentration, time, and technique of application, but also inconsistent outcome evaluation. The problem with evaluation is especially seen in in vivo studies, where a quantitative method was used only once and qualitative methods were not standardized, because such a standardization does not exist. Difference in local legal permissibility is also a major issue in terms of guidelines preparation e.g., the use of sodium perborate is not legal in the Europe. Calcium hydroxide was placed in the pulp cavity after bleaching [30] only in one case. The access cavity was restored with composite [9,27,29,30].

Taking the above into consideration, a standardized protocol for the bleaching procedure and assessment should be introduced to obtain the most reliable results.

## 5. Conclusions

This systematic review indicated that whitening of discolored teeth after REPs is achievable. The internal method was the dominant one, but due to alternative method usage shortfall, it is hard to make a comprehensive comparison. Similar to difference in bleaching agent usage observed in analyzed studies, there is a wide difference in bleaching duration. Therefore, it is not possible to make a suitable conclusion. For the creation of precise guidelines that would define the appropriate bleaching technique, material, and duration in discolored teeth after REPs, further studies are required.

## Figures and Tables

**Table 1:** The search phrases.

Database		Search Phrases
Medline [PubMed] (547)	1	(((((((((((regenerative endodontic procedures) OR regenerative endodontic treatment) OR revascularization) OR revitalization) OR Biodentine) OR mineral trioxide aggregate) OR triple antibiotic paste) OR blood) OR platelet rich fibrin) OR platelet rich plasma) OR PRP) OR PRF
	2	(discolo*) OR stain*
	3	((((bleach*) OR whiten*) OR sodium perborate) OR hydrogen peroxide) OR carbamide peroxide
	All	((((((((((((((regenerative endodontic procedures) OR regenerative endodontic treatment) OR revascularization) OR revitalization) OR Biodentine) OR mineral trioxide aggregate) OR triple antibiotic paste) OR blood) OR platelet rich fibrin) OR platelet rich plasma) OR PRP) OR PRF)) AND ((discolo*) OR stain*)) AND (((((bleach*) OR whiten*) OR sodium perborate) OR hydrogen peroxide) OR carbamide peroxide)
Scopus (734)	1	(ALL (“tooth”) OR ALL (“teeth”))
	2	(ALL (“regenerative endodontic procedures”) OR ALL (“regenerative endodontic treatment”) OR ALL (“revascularization”) OR ALL (“revitalization”) OR ALL (“Biodentine”) OR ALL (“mineral trioxide aggregate”) OR ALL (“triple antibiotic paste”) OR ALL (“blood”) OR ALL (“platelet rich fibrin”) OR ALL (“platelet rich plasma”) OR ALL (“PRP”) OR ALL (“PRF”))
	3	(ALL (discolo*) OR ALL (stain*))
	4	(ALL (bleach*) OR ALL (whiten*) OR ALL (“sodium perborate”) OR ALL (“hydrogen peroxide”) OR ALL (“carbamide peroxide”))
	All	((ALL (“tooth”) OR ALL (“teeth”))) AND ((ALL (“regenerative endodontic procedures”) OR ALL (“regenerative endodontic treatment”) OR ALL (“revascularization”) OR ALL (“revitalization”) OR ALL (“Biodentine”) OR ALL (“mineral trioxide aggregate”) OR ALL (“triple antibiotic paste”) OR ALL (“blood”) OR ALL (“platelet rich fibrin”) OR ALL (“platelet rich plasma”) OR ALL (“PRP”) OR ALL (“PRF”))) AND ((ALL (discolo*) OR ALL (stain*))) AND ((ALL (bleach*) OR ALL (whiten*) OR ALL (“sodium perborate”) OR ALL (“hydrogen peroxide”) OR ALL (“carbamide peroxide”)))
Web of Science (376)	1	ALL FIELDS: (regenerative endodontic procedures) OR ALL FIELDS: (regenerative endodontic treatment) OR ALL FIELDS: (revascularization) OR ALL FIELDS: (revitalization) OR ALL FIELDS: (Biodentine) OR ALL FIELDS: (mineral trioxide aggregate) OR ALL FIELDS: (triple antibiotic paste) OR ALL FIELDS: (blood) OR ALL FIELDS: (platelet rich fibrin) OR ALL FIELDS: (platelet rich plasma) OR ALL FIELDS: (PRP) OR ALL FIELDS: (PRF)
	2	ALL FIELDS: (discolo*) OR ALL FIELDS: (stain*)
	3	ALL FIELDS: (bleach*) OR ALL FIELDS: (whiten*) OR ALL FIELDS: (sodium perborate) OR ALL FIELDS: (hydrogen peroxide) OR ALL FIELDS: (carbamide peroxide)
	All	-
Cohrane (27)	1	(regenerative endodontic procedures OR regenerative endodontic treatment OR revascularization OR revitalization OR Biodentine OR mineral trioxide aggregate OR triple antibiotic paste OR blood OR Platelet rich fibrin OR Platelet rich plasma OR PRP OR PRF)
	2	(discolo* OR stain*)
	3	(bleach* OR whiten* OR sodium perborate OR hydrogen peroxide OR carbamide peroxide)
	All	(regenerative endodontic procedures OR regenerative endodontic treatment OR revascularization OR revitalization OR Biodentine OR mineral trioxide aggregate OR triple antibiotic paste OR blood OR Platelet rich fibrin OR Platelet rich plasma OR PRP OR PRF) in All Text AND (discolo* OR stain*) in All Text AND (bleach* OR whiten* OR sodium perborate OR hydrogen peroxide OR carbamide peroxide) in All Text

**Table 2:** Risk of bias according to the modified Methodological Index for Non-randomized Studies (MINORS) scale in in vitro studies.

	Kirchhoff et al. [22]	Yasa et al. [16]	Akbulut et al. [23]	Iriboz et al. [24]	Santos et al. [25]	Fundaoğlu Küçükekenci et al. [26]
Clear aim	2	2	2	2	2	2
Clear REPs protocol	1	1	2	1	1	1
Clear bleaching protocol	2	2	2	2	2	2
Prospective collection of data	2	2	2	2	2	2
Justification of sample size	0	2	0	0	0	2
Follow-up period appropriate to the aim of the study	2	2	2	2	2	2
Endpoints appropriate to the aim of the study	2	2	2	2	2	2
Blinded analysis	0	0	0	0	0	0
An adequate control group	0	0	0	1	2	2
Contemporary groups	0	0	0	2	2	2
Baseline equivalence of groups	2	2	2	2	2	2
Adequate statistical analyses	2	2	2	2	2	2
Total score	15	17	16	18	19	21
Study quality	fair	good	fair	good	good	good

Numbers coding: 2, reported and adequate; 1, reported but inadequate; 0, not reported.

**Table 3:** Risk of bias according to the modified Methodological Index for Non-randomized Studies (MINORS) scale in in vivo studies.

	Kim et al. [9]	Miller et al. [27]	McTigue et al. [17]	D’Mello et al. [11]	De-Jesus-Soares et al. [28]	Kahler et al. [29]	Parthiban et al. [30]	Timmerman et al. [31]	Antov et al. [32]
Clear aim	1	2	1	2	2	1	2	2	1
Clear REPs protocol	2	2	2	2	2	2	2	2	2
Clear bleaching protocol	2	1	1	2	2	1	1	2	2
Prospective collection of data	2	2	2	2	2	2	2	2	2
Justification of sample size	0	0	0	0	0	0	0	0	0
Follow-up period appropriate to the aim of the study	1	2	2	0	2	0	2	0	1
Endpoints appropriate to the aim of the study	1	1	1	2	2	1	2	1	2
Blinded analysis	0	0	0	0	0	0	0	0	0
Total score	9	10	9	10	12	7	11	9	10
Study quality	fair	fair	fair	fair	good	fair	good	fair	fair

Numbers coding: 2, reported and adequate; 1, reported but inadequate; 0, not reported.

**Table 4:** Data pertaining to analyzed in vitro studies.

Author/Year	No. of Teeth/Teeth Type	Intracanal Medicament or/and Coronal Barrier	Discoloration Yes/No	Bleaching Material	Bleaching Method	Measurement Method	Measurement Time	Bleaching Effect
Kirchhoff et al., 2015 [22]	20 human teeth	TAPM/in teeth with CA TAPM/in teeth with OA	Yes Yes	SP +DW	Material placed in the pulp chamber	Vita EasyShade Advance 4.0	1 w, 2 w, 3 w	The bleaching effect was similar between CA and OA groups
Yasa et al., 2015 [16]	40 bovine teeth	TAPM TAPD TAPA TAPC	Yes Yes Yes Yes	35% HP SP +DW	Material placed in the pulp chamber	Spectro Shade Micro	4 d, 8 d, 12 d	The whitening effect of the 35%HP was superior than SP. Discoloration caused by the TAPM and TAPC was more bleached compared to TAPD and TAPA
Akbulut et al., 2017 [23]	42 human teeth	TAPC PMTA + blood BD + blood MM-MTA + blood	Yes Yes Yes Yes	35% HP	Material placed over coronal barrier	Vita EasyShade Advance	1 w	The group with BD was more significantly whitened than the groups with PMTA and MM-MTA
Iriboz et al., 2017 [24]	85 human teeth	TAPM MP	Yes Yes	SP SP +Heat SP +Ultrasonic instrument 30 s, 29k Hz SP +Ultrasonic instrument 60 s, 28k Hz	Material placed in the pulp chamber	Vita EaSyshade	3 d, 1 w	Bleaching was observed in all groups
Santos et al., 2017 [25]	50 bovine teeth	TAPM/WMTA TAPA/WMTA DAP/WMTA CH/WMTA TAPM/GIC TAPA/GIC DAP/GIC CH/GIC CG	Yes Yes Yes Yes Yes Yes Yes Yes No	37% CP	Bleaching gel was applied inside and outside dental crowns for 45 min. Two bleaching sessions were performed at 1-week interval	Vita EasyShade VITA Classical A1–D4 Shade Guide	1 d, 8 d	No difference was found between first and second bleaching within the same group
Fundaoğlu Küçükekenci et al., 2019 [26]	120 human teeth	TAPM DAP TAPA TAPC TAPD	Yes No Yes Yes Yes	35% HP 35%HP +Nd-YAG laser irradiation	Material placed in the pulp chamber	Vita EasyShade Advance 4.0	4 d, 8 d, 12 d	The bleaching effect was superior in TAPM group than in other groups

Abbreviations: BD, Biodentine; CA, closed apices; CG, control group; CH, calcium hydroxide; CP, carbamide peroxide; d, day; DAP, double antibiotic paste; DW, distilled water; GIC, glass ionomer cement; HP, hydrogen peroxide; MP, minocycline paste; OA, open apices; PMTA, Pro Root MTA; SP, sodium perborate; TAPA, triple antibiotic paste with amoxicillin; TAPC, triple antibiotic paste with cefaclor; TAPD, triple antibiotic paste with doxycycline; TAPM, triple antibiotic paste with minocycline; w, week; WMTA, white MTA.

**Table 5:** Data pertaining to analyzed in vivo studies.

Author/Year	Patient Age	Tooth Type	Injury	Preoperative Diagnosis	Intracanal Medicament	Coronal Barrier	Discoloration Yes/No/Color	Bleaching Material	Bleaching Method	Bleaching Effect Yes/No
Kim et al., 2010 [9]	7 y	11	Uncomp. fracture	NP, SAP	TAPM	MTA	Yes Blue-greyish discoloration	SP + DW	3 × 1 w Cervical sealing with GIC 4 mm of MTA was left in canal	Yes The cervical shade improved, the tooth did not return to its original shade, patient was satisfied with the tooth whitening
Miller et al., 2012 [27]	9 y	11	Avulsion	AIP, AAP	TAPM	WMTA	Yes Grey discoloration	SP + S	1 × 1 w	Yes The patient and child were pleased with the final tooth color
McTigue et al., 2013 [17]	7 y	11	Intrusion	NP, CAA	TAPM	GMTA	Yes	Cotton pellet saturated with 35% HP for 3 min, SP + 10% HP	1 × 1 w	11 teeth were successfully bleached 3 cases could not be bleached sufficiently
	7 y	21	Intrusion	NP, CAA	TAPM	GMTA	Yes			
	7 y	21	Palatal luxation	NP, AAP	TAPM	GMTA	Yes			
	7 y	21	Avulsion	NP, AAP	TAPM	GMTA	Yes			
	10 y	22	Dens evaginatus	NP, AAP	TAPM	GMTA	Yes			
	8 y	21	Extrusion	NP, CAA	TAPM	GMTA	Yes			
	11 y	21	Extrusion	NP, AAP	TAPM	GMTA	Yes			
	7 y	21	Avulsion	NP, AAP	TAPK	WMTA	Yes			
	6 y	11	Compl. fracture	NP, AAP	TAPK	WMTA	Yes			
	9 y	21	Extrusion	NP, CAA	TAPK	WMTA	Yes			
	6 y	31	Uncomp. fracture	NP, CAA	TAPK	WMTA	Yes			
	9 y	11	Avulsion	NP, CAA	TAPK	WMTA	Yes			
	9 y	21	Avulsion	NP, CAA	TAPK	WMTA	Yes			
	8 y	11	Uncomp. fracture	NP, AAA	TAPK	WMTA	Yes			
D’Mello et al., 2017 [11]	7 y	11	Uncomp. fracture	NP, APP, FC	Odontopaste	Surgicel WMTA	Yes Grey discoloration	SP + SW	1 × 2 w Cervical sealing with GIC 3 mm of WMTA was left in canal	Yes The tooth achieved a natural tooth color similar to the adjacent tooth
De-Jesus-Soares et al., 2018 [28]	8 y	11 21	Compl. fracture Uncomp. fracture	NP NP	TAPM CH+2% chlorhexidine gel	MTA MTA	Yes Yes	SP + DW	2 × 1 w Cervical sealing with Coltosol. MTA was left in canal	Yes Yes Both teeth were similar to the color of the other teeth
Kahler et al., 2018 [29]	11 y	35	-	NP, CAA	TAPA	WMTA	Yes	35% HP	Internal bleaching	Yes
		45	-	NP, APP	TAPA	WMTA	Yes	35% HP	Internal bleaching	Yes Both teeth were successfully bleached
Parthiban et al., 2018 [30]	14 y	21	Crown fracture	NP, AAP	TAPM	PRF MTA	Yes Blue-greyish discoloration	SP + 30% HP	Cervical sealing with GIC CH was placed in the access cavity after bleaching	Yes Satisfactory results were achieved
Timmerman et al., 2018 [31]	12 y	45	IP	APP	-	Surgicel, WMTA	Yes greyish-blue discoloration	SP + DW	1 × 3 w Cervical sealing with Cavit W. WMTA was removed from canal	Yes Patient was satisfied with the tooth color after bleaching
Antov et al., 2019 [32]	15 y	11	Uncomp. fracture	NP, CAP	DAP	WMTA	Yes	10% CP	1 × 2 w (4 h per day) Internal/external bleaching Cervical sealing with GIC Part of WMTA was left in canal	Yes Tooth had lightened from C3 to B1 shade
	9 y	22	-	NP, CAP	DAP	PC	Yes	10% CP	2 × 2 w (4 h per day) Internal/external bleaching cervical sealing with GIC Part of PC was left in canal	Yes There was minimal improvement of the shade, patient was no longer concerned about the discoloration
	14 y	21	Uncomp. Fracture	NP, CAP	DAP	PC	Yes	10%CP	1 × 2 w (4 h per day) External bleaching	Yes Improvement of shade was noted, the patient was not fully satisfied with the shade

Abbreviations: AAA, acute alveolar abscess; AAP, asymptomatic apical periodontitis; AIP, asymptomatic irreversible pulpitis; APP, acute periapical periodontitis; CAA, chronic alveolar abscess; CAP, chronic apical periodontitis; CH, calcium hydroxide; Compl. Fracture, complicated crown fracture; CP, calcium perborate; DAP, double antibiotic paste; DW, distilled water; FC, facial cellulitis; GIC, glass ionomer cement; GMTA, grey MTA; h, hours; HP, hydrogen peroxide; IP, irreversible pulpitis; MTA, mineral trioxide aggregate; NP, necrotic pulp; PC, portland cement; PRF, platelet rich fibrin; S, saline; SAP, symptomatic apical periodontitis; SP, sodium perborate; SW, sterile water; TAPA, triple antibiotic paste with amoxicillin; TAPK, triple antibiotic paste with clindamycin; TAPM, triple antibiotic paste with minocycline; Uncomp. Fracture, uncomplicated crown fracture; w, week; WMTA, white MTA; y, year.
